# Structural Stabilities and Transformation Mechanism of Rhynchophylline and Isorhynchophylline by Ultra Performance Liquid Chromatography/Time-of-Flight Mass Spectrometry (UPLC/Q-TOF-MS)

**DOI:** 10.3390/molecules200814849

**Published:** 2015-08-14

**Authors:** Zhen-Feng Wu, Ya-Qi Wang, Na Wan, Gang Ke, Peng-Fei Yue, Hao Chen, Juan-Juan Zhan, Ming Yang

**Affiliations:** 1 Key Laboratory of Modern Preparation of Traditional Chinese Medicine, Ministry of Education, Jiangxi University of Traditional Chinese Medicine, Nanchang 330004, China; E-Mails: wangyaqi_3@163.com (Y.-Q.W.); wanna988@163.com (N.W.); kg_rgl@126.com (G.K.); ypfpharm@126.com (P.-F.Y.); 15570370525@163.com (J.-J.Z.); tcmmingyang@163.com (M.Y.); 2 Luzhou People’s Hospital, Luzhou 646100, China; 3 Affiliated Hospital of Jiangxi University of Traditional Chinese Medicine, Nanchang 330004, China; E-Mail: 13707096286@163.com

**Keywords:** rhynchophylline, isorhynchophylline, conversion rate, stability, UPLC-Q-TOF-MS

## Abstract

To reveal the structural stabilities and transformation mechanism of rhynchophylline (RIN) and isorhynchophylline (IRN), HPLC and UPLC-Q-TOF-MS method were developed for the qualitative and quantitative analysis of the conversion rate. The method was validated for linearity, inter- and intra-day precisions, repeatability and stability. All the quantitative determination method validation results were satisfactory. Under the optimized chromatographic conditions, the effect of various heat temperatures, retention time, and solvent polarities on conversion rate and equilibrium were systematically investigated for the first time. Besides, a model relating the retention yield value and time-temperature was built to predict the *t*_0.5_ and *Ea* of the conversion rate by the Arrhenius equation. The experimental results proved to be in good accordance with the predicted values. Furthermore, UPLC-Q-TOF-MS analysis was performed to verify the transformation mechanism and provide valuable information for stability analysis of the conversion products.

## 1. Introduction

**Figure 1:**
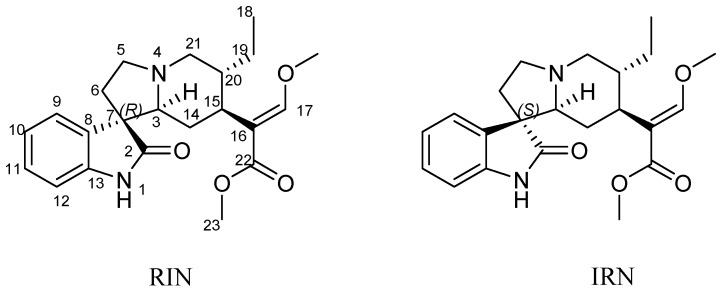
The chemical structure of rhynchophylline (RIN) and isorhynchophylline (IRN).

*Uncaria rhynchophylla* (Miq.) known as Gou-teng in Chinese, is one of the most famous herbal resources, extensively used as a traditional medicine in China, India, Korea, and other Southeast Asian countries for prevention and treatment of hypertension, cardiac arrhythmia, anxiety, convulsions and epilepsy [1,2]. Furthermore, it represents a promising new way to treat Alzheimer’s disease [3,4]. The main active compounds in *U. rhynchophylla* are believed to be indole alkaloids, and rhynchophylline (RIN) and isorhynchophylline (IRN) are the most important and major alkaloids in *U. rhynchophylla*, showing manifold activities [5,6,7]. Therefore, RIN and IRN are often used as chemical markers for the quality control of *U. rhynchophylla*. However, according to the research, the analysis performance varied between lots, and widely between similar methods, which may seriously affect the subsequent quality control of *U. rhynchophylla*. RIN and IRN are a pair of diastereoisomers (Figure 1), which is liable to transform mutually due to their twisted conformation [8]. Besides, our previous study proved the existence of a tautomerism phenomenon between RIN and IRN.

In order to obtain information to control the quality of the preparation of *U. rhynchophylla* and provide valuable information for industrial purposes, the stabilities and transformation mechanism of RIN and IRN were investigated. To our knowledge, there are no studies about the stability and conversion rates between RIN and IRN. The effects of various temperatures, reaction times, and solvent polarities were investigated. The Arrhenius equation was used to build a model linking the conversion rate and time-temperature, and to predict the *t*_0.5_ and *Ea* of the conversion rate. Furthermore, UPLC-Q-TOF-MS analysis was performed to verify the transformation mechanism and provide valuable information for the analysis of the stability of the conversion products.

## 2. Results and Discussion

### 2.1. HPLC Analysis

To achieve a reliable resolution of the analytes, several mobile phase additives including formic acid and ammonium acetate were tested. It was found that the good signal intensity, resolution and peak shape were obtained when the concentrate of ammonium acetate reached 0.01 mol∙L^−1^, then the value of pH was adjusted to 8.0 by ammonia. A few different columns (Welch Xtimate C_18_, Phenomenex Gemini C_18_ and Agilent Zorbax C_18_) were tested, The Phenomenex Gemini C_18_ column was finally selected as the most suitable choice for HPLC analysis of RIN and IRN*.* To obtain a sufficiently large number of detectable peaks in the chromatographic fingerprints, DAD full scan was used to acquire all the main peaks and finally 245 nm was selected as the detection wavelength. There was no sharp effect of the column temperature (25–30 °C) on either the peak symmetry or the resolution of the eluted peaks, so 30 °C was selected as the optimum column temperature. Under these optimum conditions, all the studied chemical constituents were well separated from each other by HPLC.

### 2.2. Effect of Time-Temperature on Rates and Equilibria

**Figure 2:**
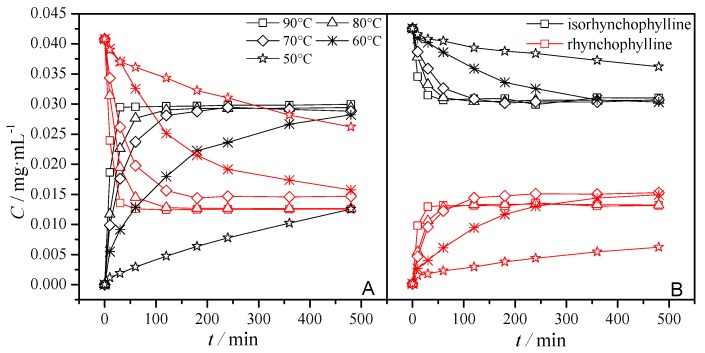
Effect of time-temperature conditions on the conversion rates of rhynchophylline (**A**) and isorhynchophylline (**B**).

Thermal processing had a positive effect on the conversion rates of both RIN and IRN. HPLC was adopted to determine the concentrations and structural changes between the RIN and IRN at different temperatures and time points (10, 30, 60, 120, 180, 240 and 480 min). The results (Figure 2) clearly indicated that RIN and IRN tend to transform into each other, and the rate was increased with temperature. This phenomenon might be caused by a retro-Mannich type reaction, which would cause the configuration inversion of spirooxindole alkaloids. With the increase of reaction time, the transformation could reach equilibrium and thus was not readily affected by changes in the time of reaction. A similar result has been reported by Laus [8].

For the conversion rate of RIN Figure 2A showed that the residual value decreased dramatically as the heating temperature increased from 50 °C to 90 °C. There was a 4.4% and 41.4% decrease in the residual value of RIN after hold 10 min at 50 °C and 90 °C, respectively. This also indicated that a higher temperature was more effective for the equilibrium of the conversion reaction. After reacting for nearly 50 min at 90 °C, 100 min at 80 °C, or 150 min at 70 °C, the conversion could reach equilibrium where there was almost 70% RIN converted into IRN, and only 30% RIN was retained, so the ratio of RIN to IRN was 7:3.

For the conversion rate of IRN, Figure 2B showed that there was a 2.3% and 18.8% decrease in the residual value of IRN after holding for 10 min at 50 °C and 90 °C, respectively. After reaction for nearly 50 min at 90 °C, 100 min at 80 °C, or 150 min at 70 °C, the conversion could reach equilibrium where there was only 30% RIN converted into IRN, almost 70% RIN was retained, therefore the ratio of RIN to IRN was 7:3 as well.

### 2.3. Kinetic Analysis

To study the conversion rate of RIN and IRN, the experimental results were analyzed by the Arrhenius equation. From Table 1, the values for the coefficient of determination (*r*) for the kinetic parameters under different temperature were greater than 0.9, which implied that over 90% of the variation for the process efficiency could be explained by these models. The closer *R*^2^ is to 1, the better the prediction models fits the actual data. At a temperature of 90 °C, the *t*_0.5_ of RIN and IRN were only 0.81 and 3.10 days, respectively, which increased with the decrease of the heating temperature. It was suggested that, to a certain degree, increased reaction time and temperature would accelerate the conversion rate of RIN and IRN. The thermal transformation of RIN and IRN complied with first-order reaction kinetics. Moreover, the *Ea* values in the prediction model were 87.01 and 81.26 kJ∙mol^−1^, respectively, which could be calculated according to Arrhenius equation. It was suggested that RIN and IRN were liable to convert into each other. Low temperatures and short reaction times during heat treatment were beneficial to maintaining the stability of both compounds.

**Table 1:** Kinetic parameters of rhynchophylline and isorhynchophylline.

Heating Temperature/°C	Rhynchophylline			Isorhynchophylline		
	K/h^−1^	*r*	*t*_0.5_/d	K/h^−1^	*r*	*t*_0.5_/d
50	0.0009	0.9974	32.08	0.0003	0.9939	96.25
60	0.0037	0.9964	7.80	0.0012	0.9874	24.06
70	0.0078	0.9576	3.70	0.0025	0.9267	11.55
80	0.0172	0.9743	1.68	0.0050	0.9463	5.78
90	0.0356	0.9854	0.81	0.0093	0.9184	3.10

### 2.4. Effect of Solvent on Rates and Equilibria

**Figure 3:**
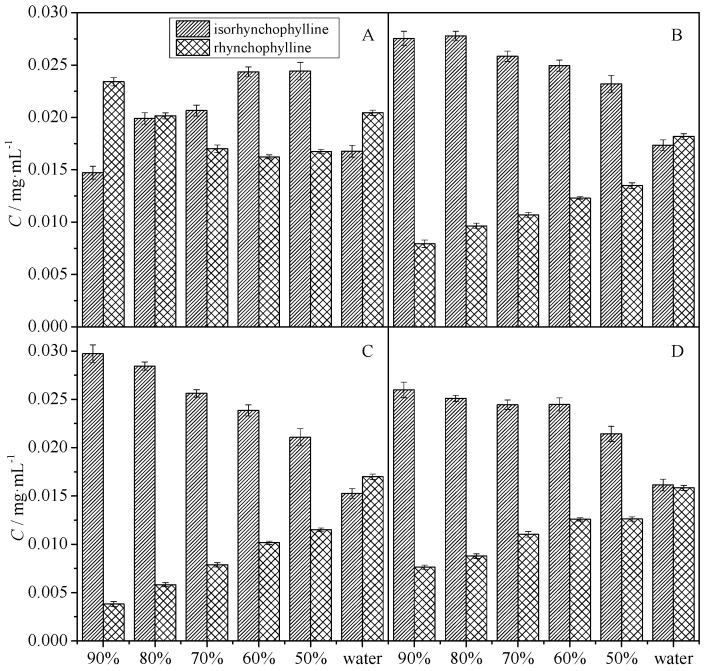
Effect of different solvent on the rate of conversion of RIN and IRN. (**A**) RIN reaction for 30 min at 80 °C; (**B**) RIN reaction for 300 min at 80 °C; (**C**) IRN reaction for 30 min at 80 °C; (**D**) IRN reaction for 300 min at 80 °C.

In general, the nature of the solvent is considered an important parameter for heat stability because it might affect the absorption of energy and the solubility of the target ingredients [9]. Owing to the polarity of RIN and IRN, and the toxicity of some solvents, ethanol was chosen as the solvent. As seen in Figure 2, after reaction for 100 min at 80 °C, the conversion rate could reach equilibrium. Therefore, in order to ensure a complete reaction, we fixed the heat temperature at 80 °C and the end of the reaction at 300 min in this study, and the impact of different ethanol concentrations (90%, 80%, 70%, 60%, 50% and 100% water) on the conversion rate and equilibrium is shown in Figure 3.

Figure 3A shows that the residual value of RIN decreased dramatically within the first 30 min. After reaction for 300 min at 80 °C, the conversion could reach equilibrium. From Figure 3B,D, it could be concluded that the final ratio of RIN to IRN changes depending on the proportion of solvent. The final ratio value increased with the decrease of ethanol concentration. Besides, despite the fact different proportions of RIN and IRN were used, some kinds of equilibrium would ultimately result due to the solvent.

### 2.5. Transformation Mechanism Analysis

#### 2.5.1. UPLC-Q-TOF-MS Conditions

The effect of different mobile phase compositions on the chromatographic separation was examined. Acetonitrile-water possessed higher resolution and better peak shape than methanol-water. Different flow rates of 0.2, 0.3 and 0.4 mL∙min^−1^ were also investigated, and 0.3 mL∙min^−1^ was selected as the preferable one to get the best resolution. In the process of gradient optimization, gradient time, gradient procedure and initial composition of the mobile phase were all taken into consideration. The final gradient procedure is described in Section 3.6. UPLC-Q-TOF-MS analysis.

#### 2.5.2. Diagnostic Fragmentation ions of RIN and IRN

**Figure 4:**
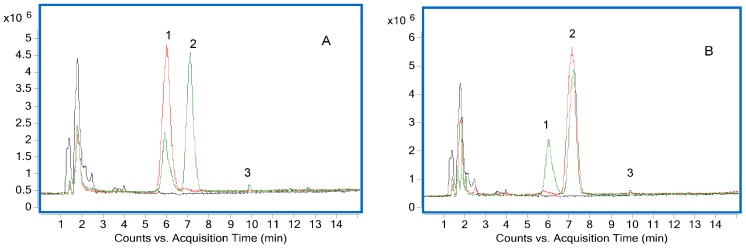
Total ion chromatogram in positive ion mode of the conversion products of rhynchophylline (**A**) and isorhynchophylline (**B**). Reference standards are shown in red, transformation products in green and solvent blank in black.

The chemical structures of RIN and IRN correspond to a pair of diastereoisomeric C2 oxindole alkaloids, whose only difference is the configuration at C7, (Figure 1). The positive ion mode was the most suitable for the analysis of the alkalescent molecule. As seen in Figure 4, RIN and IRN could mutually interconvert, and eventually achieve a balance determined by the polarity of the solvent and isomer ratio yield value. By comparing to the solvent blank, ions in the first three minutes could be deducted as a part of background. Clearly, the major conversion product was RIN or IRN, nevertheless, a small peak at 9.8 min appeared repeatedly both in Figure 4A,B, which might be recognized as the possible intermediate in the transformation process. To further verify the transformation mechanism and provide more information for the identification of this possible intermediate in the transformation process, the fragmentation regularity of C2 oxindole alkaloid isomers (RIN and IRN) was studied according to the UPLC-Q-TOF-MS data.

**Figure 5:**
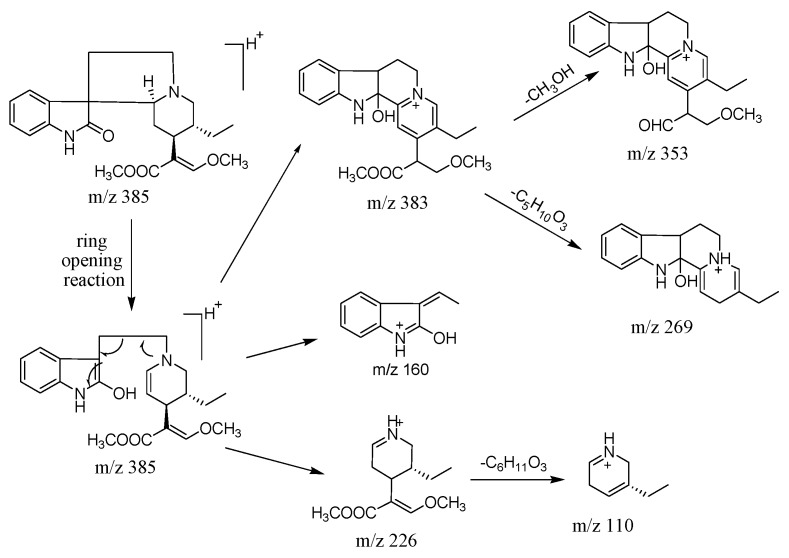
The proposed fragmentations pathways of RIN and IRN.

As seen in Table 2, the fragment ions of RIN and IRN were similar, which is not unexpected since compounds with the same chemical skeleton should always produce similar MS fragment ions, and the fragmentation pathways were somewhat coherent [10]. The MS spectra of the three peaks are shown in The Supplementary Materials (Figures S1–S3). The retention time of RIN was 6.1 min, and 7.2 min for IRN, with a maximum UV absorption at 245 nm as well. In positive ion mode, a molecular ion at *m*/*z* 385 [M + H]^+^ was found in the MS spectrum. The structures of RIN and IRN both have methyl groups attached to the core molecules by ester bonds which were readily dissociated, so characteristic fragment ions at *m*/*z* 353 [M + H-CH_3_OH]^+^ were formed by the losses of a methanol molecule (32 amu). Fragmentation of the ion at *m*/*z* 269 [M + H-C_5_H_8_O_3_]^+^ arose from the loss of a unit with a molecular weight of 116 amu. The fragment ions with the highest abundance at *m*/*z* 160 was formed by the transfer of a hydrogen atom from a five membered nitrogen-containing heterocyclic to the oxy group of C2, which then causes the band cleavage of the N4 and C5 [11]. A proposed fragmentation pathway of RIN and IRN was shown in Figure 5.

**Table 2:** The fragment ions of characteristic peaks by UPLC-Q-TOF analysis.

No.	Molecular Formula	[M + H]^+^/*m/z*	Error/ppm	Fragmentions/*m/z*	Identication
Peak 1	C_22_H_28_N_2_O_4_	385.2116	1.95	385.2116, 353.1859, 269.1648, 160.0757, 110.0964	RIN
Peak 2	C_22_H_28_N_2_O_4_	385.2119	0.82	385.2119, 353.1858, 267.1490, 160.0756, 108.0808	IRN
Peak 3	C_22_H_2__6_N_2_O_4_	383.1956	-	383.1961, 353.1851, 269.1643, 160.0756, 110.0963	-

**Figure 6:**
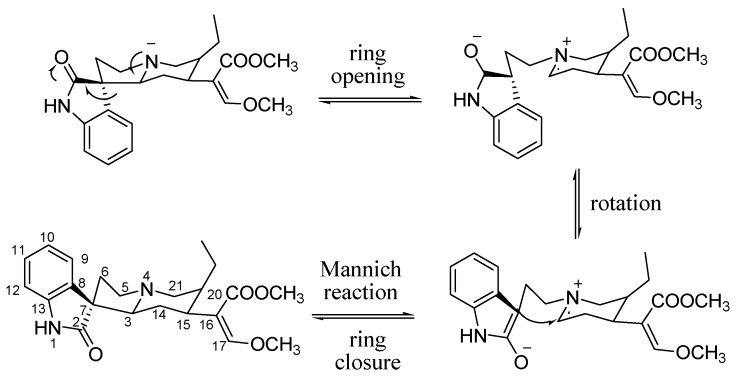
The mutual transformation between RIN and IRN.

From Figure 5, obviously the ions at *m*/*z* 385, 383, 353, 269, 160 and 110 were diagnostic fragmentation ions of the C2 oxindole alkaloid isomers (RIN and IRN). These ions might be used to rapidly identify a class of compound with similar structures [12]. For peak 3, ions at *m*/*z* 353, 269, 160 and 110 were observed in its MS spectrum in positive ion mode, which might recognized as the possible intermediate in the transformation process. Besides, ion at *m*/*z* 383 was observed, which was coherent with the transformation process of losing two ions after a ring opening reaction. The mutual transformation between RIN and IRN is shown in Figure 6 [11,12].

## 3. Experimental Section

### 3.1. Materials

Reference samples of RIN (No. 1266-070214, ≥98.0% purity) and IRN (No. Y13-130012, ≥98.0% purity) were purchased from National Engineering Research Center of Manufacturing Technique of Solid Preparations of Chinese medicine (Nanchang, China). Methanol (chromatographic grade), acetonitrile (chromatographic grade), ethanol (chromatographic grade), ammonium acetate (analytical grade), and ammonia (analytical grade) were obtained from Sinopharm Chemical Reagent Co., Ltd (Shanghai, China).

### 3.2. Apparatus

An Agilent 1260 HPLC equipped with a DAD ultraviolet detector (Agilent Technologies, Santa Clara, CA, USA) was used for HPLC analysis. A Kinetex C_18_ column (100 × 2 mm, 2.6 μm) and a Gemini C_18_ column (250 × 4.6 mm, 5 μm, Phenomenex, Torrance, CA, USA) were used for the UPLC-Q-TOF-MS and HPLC analysis, respectively. An Agilent 6538 UPLC-ESI-Q-TOF-MS system equipped with an on-line degasser, dual gradient pump, autosampler and column oven was used for MS analysis.

### 3.3. Preparation of Rhynchophylline and Isorhynchophylline Solutions

The reference compounds of RIN and IRN were accurately weighed and were dissolved in 70% ethanol-water solution at 1.020 and 1.004 mg∙mL^−1^, respectively (stock solutions). An aliquot of the stock solution (2.0 mL) was transferred to a 50 mL volumetric flask, diluted with 70% ethanol water solution to volume, and mixed well as test solution for the heat stability analysis. Each test solution (1 mL) was transfered to a 5 mL centrifuge tube, well-sealed and weighed. Each sample was then heated under various temperatures and reaction times using a water bath. Besides, stock solution (1 mL) was transferred to a 25 mL volumetric flask, diluted individually with 50%, 60%, 70%, 80% and 90% ethanol-water solution to volume, and mixed well as test solution for the solvent stability analysis. The stock solutions were also diluted to appropriate concentrations for establishment of calibration curves for HPLC analysis. Each test was performed in triplicate.

### 3.4. HPLC Conditions

Determination the residual value of RIN and IRN by various processing methods was analyzed by the HPLC method. The chromatographic elution system consisted of methanol (solvent A) and water with 0.01 mol∙L^−1^ ammonium acetate buffer solution (pH = 8.0) (solvent B) [13]. Separation was performed using 60% A as mobile phase at a flow rate of 1.0 mL∙min^−1^. The detection wavelength was 245 nm. The column temperature was maintained at 30 °C and the injection volume was 20 μL.

### 3.5. Calibration and Method Validation

Table 3 provides the validation parameters including calibration curves and linearity ranges. The correlation coefficients were both higher than 0.9995, which is sufficient to ensure successful quantitative application in routine analysis. The intra-day precision was tested six times within a day, and the inter-day precision was obtained from six determinations on two consecutive days. The intra-day precision of the two constituents was less than 1%, and the inter-day precision was less than 3%, as shown in Table 3. Repeatability was tested by studying five parallel samples (80 °C, 100 min). RSD values of the peak areas of RIN and IRN were 1.41% and 1.92%, respectively (Table 3). Stability was analyzed by repeated tests of the sample solutions at different storage time for 12 h (0, 1, 3, 6, 9, 12 h). The RSD values were 1.0% and 1.7% for RIN and IRN, respectively (Table 3). All the standard solutions of various concentrations were stored at 4 °C until assayed. The results implied the HPLC method was reliable for the quantitative analysis of RIN and IRN.

**Table 3:** Calibration parameters of the HPLC analysis for RIN and IRN.

No.	Regression Equation	Linear Range/μg	*R^2^*	Precision (%)		Repeatability/%	Stability/%
				Intra-Day	Inter-Day		
RIN	y = 2748x − 14.327	0.0638–2.5500	0.9999	0.39	1.83	1.41%	1.0%
IRN	y = 3448.3x − 14.773	0.0628–2.5100	0.9999	0.79	2.35	1.92%	1.7%

### 3.6. UPLC-Q-TOF-MS Conditions

The fragment ions of RIN and IRN were analyzed by the UPLC-Q-TOF-MS method. The gradient elution system consisted of acetonitrile (solvent A) and water (solvent B). Separation was achieved using the following gradient steps: 0–15 min, 30% A–60% A. The flow rate of the mobile phase was set at 0.3 mL∙min^−1^, the column temperature was maintained at 30 °C. Ultrahigh pure helium (He) and high purity nitrogen (N_2_) were used as collision and nebulizer gas, respectively. The optimized parameters in positive ion modes were as follows: fragmentor voltage, 120 V; skimmer voltage, 65 V; OCT 1 RF Vpp voltage, 750 V; capillary voltage, 4000 V; capillary temperature, 350 °C; atomizing pressure: 40 psi; dry nitrogen flow rate: 10 L∙min^−1^; collision energy: 30 eV. Spectra were recorded in the range of *m*/*z* 100–1000 for full scan data.

### 3.7. Statistical Analysis

All data were the mean value from the three samples in each sampling point and statistically analyzed by SPSS version 18. Kinetic analysis of the values from different time and temperature were calculated using the following two formulas.

(1) $$\ln K=\ln A-\frac{E_{a}}{RT}$$

(2) $$t_{0.5}=\frac{0.693}{K}$$

where *K* is the reaction rate constants, *E_a_* is the apparent activation energy (kJ∙mol^−1^), A is the frequency factor, *T* is the absolute temperature (K), and *R* is the gas constant (8.3144 × 10^−3^ kJ∙mol^−1^).

## 4. Conclusions

The effects of various heating temperatures, times, and solvent polarities on the interconversion of RIN and IRN were investigated systematically. The Arrhenius equation was used to build a model linking the conversion rate and time-temperature, and to predict the *t*_0.5_ and *Ea* of the conversion rate. The experimental results showed good agreement with the predicted values. Furthermore, UPLC-Q-TOF-MS analysis was performed to verify the transformation mechanism and provide valuable information for stability analysis of the conversion products. Based on the obtained data, the heat stability and conversion rate between RIN and IRN were demonstrated via kinetic analysis for the first time.

## Supplementary Materials

Supplementary materials can be accessed at: http://www.mdpi.com/1420-3049/20/08/14849/s1.
