# Supplementary material for: Structural Stabilities and Transformation Mechanism of Rhynchophylline and Isorhynchophylline by Ultra Performance Liquid Chromatography/Time-of-Flight Mass Spectrometry (UPLC/Q-TOF-MS)
Source: Molecules. 2015 Aug 14;20(8):14849–59. doi: 10.3390/molecules200814849 (PMC6331816; doi:10.3390/molecules200814849)
Supplement: Supplementary file 1 [file molecules-20-14849-s001.pdf]

Supplementary Materials

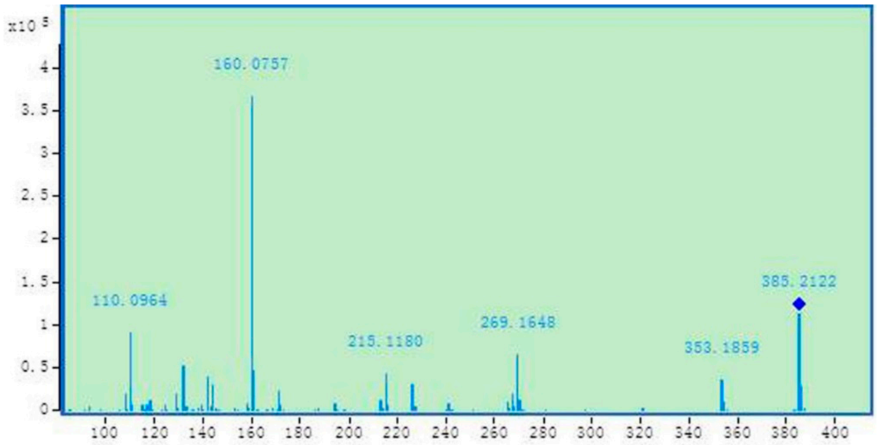

Figure S1. The MS spectra of rhynchophylline.

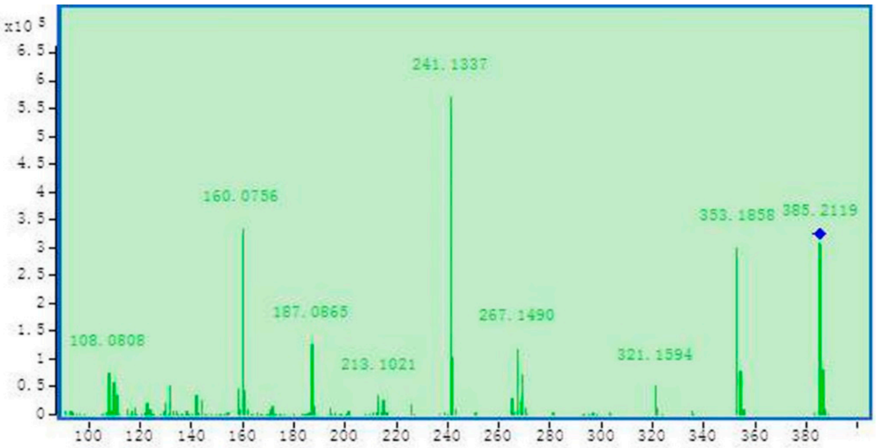

Figure S2. The MS spectra of isorhynchophylline.

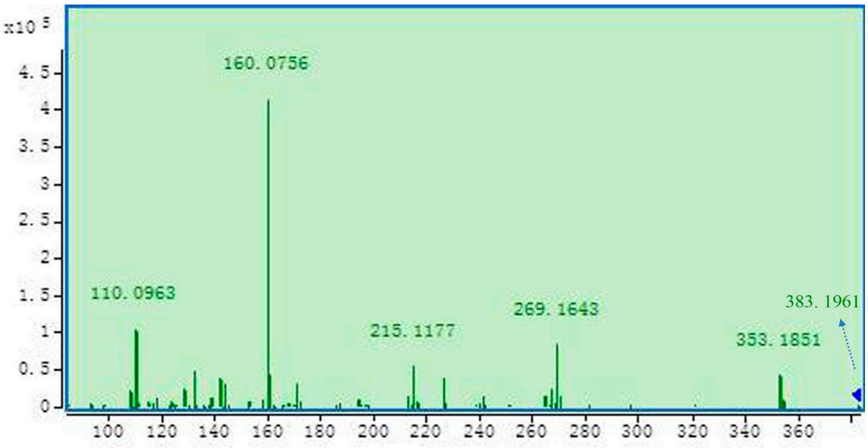

Figure S3. The MS spectra of the possible intermediate.
